# Predicting X-ray diffuse scattering from translation–libration–screw structural ensembles

**DOI:** 10.1107/S1399004715007415

**Published:** 2015-07-28

**Authors:** Andrew H. Van Benschoten, Pavel V. Afonine, Thomas C. Terwilliger, Michael E. Wall, Colin J. Jackson, Nicholas K. Sauter, Paul D. Adams, Alexandre Urzhumtsev, James S. Fraser

**Affiliations:** a Department of Bioengineering and Therapeutic Sciences, University of California San Francisco, San Francisco, CA 94158, USA; b Physical Biosciences Division, Lawrence Berkeley National Laboratory, Berkeley, CA 94720, USA; c Bioscience Division, Los Alamos National Laboratory, Los Alamos, NM 87545, USA; d Computer, Computational, and Statistical Sciences Division, Los Alamos National Laboratory, Los Alamos, NM 87545, USA; e Research School of Chemistry, Australian National University, Canberra, ACT 2601, Australia; f Department of Bioengineering, University of California Berkeley, Berkeley, CA 94720, USA; g Centre for Integrative Biology, Institut de Génétique et de Biologie Moléculaire et Cellulaire, CNRS–INSERM–UdS, 1 Rue Laurent Fries, BP 10142, 67404 Illkirch, France; h Faculté des Sciences et Technologies, Université de Lorraine, BP 239, 54506 Vandoeuvre-les-Nancy, France

**Keywords:** diffuse scattering, TLS, correlated motion, structural ensemble, structure refinement

## Abstract

Identifying the intramolecular motions of proteins and nucleic acids is a major challenge in macromolecular X-ray crystallography. Because Bragg diffraction describes the average positional distribution of crystalline atoms with imperfect precision, the resulting electron density can be compatible with multiple models of motion. Diffuse X-ray scattering can reduce this degeneracy by reporting on correlated atomic displacements. Although recent technological advances are increasing the potential to accurately measure diffuse scattering, computational modeling and validation tools are still needed to quantify the agreement between experimental data and different parameterizations of crystalline disorder. A new tool, *phenix.diffuse*, addresses this need by employing Guinier’s equation to calculate diffuse scattering from Protein Data Bank (PDB)-formatted structural ensembles. As an example case, *phenix.diffuse* is applied to translation–libration–screw (TLS) refinement, which models rigid-body displacement for segments of the macromolecule. To enable the calculation of diffuse scattering from TLS-refined structures, *phenix.tls_as_xyz* builds multi-model PDB files that sample the underlying T, L and S tensors. In the glycerophos­phodiesterase GpdQ, alternative TLS-group partitioning and different motional correlations between groups yield markedly dissimilar diffuse scattering maps with distinct implications for molecular mechanism and allostery. These methods demonstrate how, in principle, X-ray diffuse scattering could extend macromolecular structural refinement, validation and analysis.

## Abstract 2

A method of simulating X-ray diffuse scattering from multi-model PDB files is presented. Despite similar agreement with Bragg data, different translation–libration–screw refinement strategies produce unique diffuse intensity patterns.

## 1. Introduction   

Protein flexibility is essential for enzymatic turnover, signaling regulation and protein–protein interactions (Fraser & Jackson, 2011 ▸). The motions enabling these functions span length scales from a few angstroms to many nanometres and include transitions between side-chain rotamers (Fraser *et al.*, 2009 ▸), loop openings and closings (Qin *et al.*, 1998 ▸; Williams *et al.*, 2014 ▸) and rigid-body subunit rotations (Korostelev & Noller, 2007 ▸). Multiple crystal structures are routinely compared to identify these motions and to derive hypotheses about the role of correlated motions in executing protein function. However, if only a single crystal form is available, evidence of concerted motion must be extracted from the spread in the electron density.

Extracting this information is possible because protein conformational heterogeneity across unit cells in space and within unit cells during the X-ray exposure time leads to an ensemble-averaged electron-density map. Atomic vibrations are commonly fitted with individual *B* factors, which describe the electron-density distribution as a continuous isotropic Gaussian envelope around a central location and predominantly encompass disorder from thermal motion. Discrete conformational heterogeneity and crystal-packing defects can be described as ensembles of structural models with partial occupancy (Burnley *et al.*, 2012 ▸; Rader & Agard, 1997 ▸; Gros *et al.*, 1990 ▸; van den Bedem *et al.*, 2009 ▸; Levin *et al.*, 2007 ▸; Wall, Clarage *et al.*, 1997 ▸). If high-resolution diffraction data are available, anisotropic directionality can be added to *B* factors by modeling a Gaussian distribution along each real-space axis, yielding an ellipsoid that shows the predominant direction of the electron density.

However, the large number of parameters required for anisotropic *B*-factor refinement renders it inaccessible for most macromolecular diffraction experiments. Translation–libration–screw (TLS) modeling, introduced by Schomaker & Trueblood (1968 ▸), can describe concerted, rigid-body displacement of groups of atoms (for a comprehensive review, see Urzhumtsev *et al.*, 2013 ▸). In TLS refinement, the target protein is segmented into independent rigid bodies that undergo small translations (‘vibrations’) and rotations (‘librations’). The anisotropic displacement of TLS refinement can be fully described with 20 parameters per rigid body, with each rigid body potentially containing many atoms. This small number of parameters compares favorably with the six parameters per atom demanded by individual anisotropic *B*-factor refinement and allows grouped anisotropic *B* factors to be modeled at medium- to low-resolution ranges. TLS refinement often leads to better agreement between observed and calculated structure factors, as measured by decreasing *R*_free_ values. The potential for improved statistics when relatively few observations are available has positioned TLS as a general refinement technique: roughly 22% of the structures deposited in the Protein Data Bank (PDB; Bernstein *et al.*, 1977 ▸; Berman *et al.*, 2000 ▸) employ TLS refinement in some form. TLS refinement is a component of many major structural refinement programs such as *REFMAC* (Murshudov *et al.*, 2011 ▸; Winn *et al.*, 2001 ▸), *BUSTER-TNT* (Bricogne, 1993 ▸; Bricogne *et al.*, 2011 ▸) and *phenix.refine* (Afonine *et al.*, 2012 ▸). These programs can select TLS groups automatically, based on biochemical intuition or with the assistance of external web servers (Painter & Merritt, 2006*a* ▸,*b* ▸).

**Figure 1:**
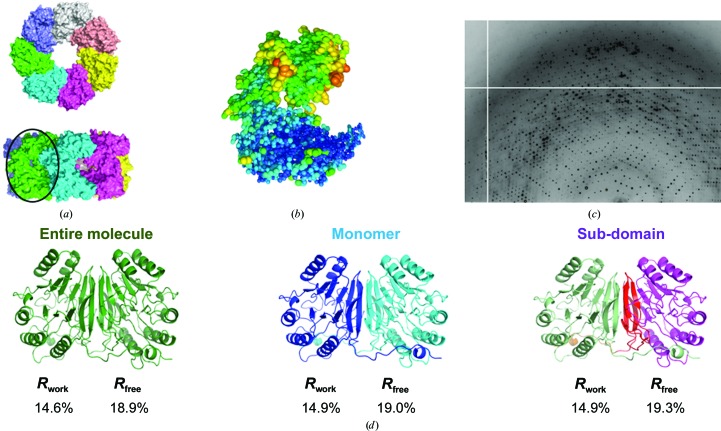
TLS refinement suggests macromolecular motions linked to function. (*a*) Top and side view of GroEL. Each color denotes a unique chain. (*b*) TLS refinement of GroEL subunits reveals a ‘tilting’ motion around the center of the subunit. (*c*) GpdQ diffraction image showing significant diffuse scattering features. (*d*) Refinement of GpdQ fails to produce substantial changes in *R*_work_ and *R*_free_ values between alternate TLS groups. TLS refinement significantly improves the overall *R*_free_ (23.1% pre-TLS).

TLS refinement naturally suggests concerted structural motions, which can be assigned biological significance and subsequently tested with additional experiments. Visualization programs such as *TLSViewer* (Painter & Merritt, 2005 ▸) can convert the T, L and S tensors into a description of domain-scale mechanical motions, and molecular-graphics programs such as *Chimera* (Pettersen *et al.*, 2004 ▸), *Coot* (Emsley & Cowtan, 2004 ▸) or *PyMOL* (DeLano, 2002 ▸) can be used to visualize the resulting anisotropic ellipsoids. For example, TLS refinement of the large multi-protein complex GroEL revealed subunit tilting that may play a role in transmitting conformational changes upon GroES or nucleotide binding (Chaudhry *et al.*, 2004 ▸; Figs. 1 ▸ *a* and 1 ▸ *b*). Similarly, TLS modeling of the ribosome structure implied a ‘ratcheting’ rotation of the 50S and 30S subunits around the peptidyltransferase center during tRNA translocation (Korostelev & Noller, 2007 ▸).

A potential complication of TLS refinement is that there is no information regarding correlations between groups; thus, many different rigid-body arrangements can result in equivalent improvement of refinement statistics (Moore, 2009 ▸; Tickle & Moss, 1999 ▸). The inability to discriminate among alternate TLS models stems from the exclusive usage of Bragg diffraction data in model refinement. Because Bragg data report on electron density averaged across all unit cells, there may be several models of correlated structural displacement that fit the density equally well. Thus, TLS refinement might improve the modeled electron density but incorrectly describe the correlated motion that occurs in the crystal during the diffraction experiment. Drawing on additional sources of information such as patterns of steric clashes (van den Bedem *et al.*, 2013 ▸), NMR spectroscopy (Ruschak & Kay, 2012 ▸) or mutational analysis (Fraser *et al.*, 2009 ▸) can be used to distinguish competing models of correlated motion between nonbonded atoms.

An additional, yet rarely used, data source that can discriminate between these models is X-ray diffuse scattering from protein crystals, which results from correlated variation in the electron-density distributions (Phillips *et al.*, 1980 ▸; Chacko & Phillips, 1992 ▸; Faure *et al.*, 1994 ▸; Mizuguchi *et al.*, 1994 ▸; Clarage & Phillips, 1997 ▸). This variation breaks from the theoretical ‘perfect’ crystal lattice, leading to diffraction outside of the regions of reciprocal space predicted by Bragg’s law. The theoretical relationship between conformational heterogeneity within unit cells and diffuse scattering has been available for decades (Guinier, 1963 ▸; Amorós & Amorós, 1968 ▸), and small-molecule crystallographers have used diffuse scattering data in refinement and model validation (Welberry & Butler, 1994 ▸; Estermann & Steurer, 1998 ▸; Michels-Clark *et al.*, 2013 ▸). 

The potential of macromolecular diffuse scattering to break the degeneracy within refinement methods such as TLS, including information about the location and length scale of macromolecular disorder, has long been recognized (Thüne & Badger, 1995 ▸; Pérez *et al.*, 1996 ▸; Héry *et al.*, 1998 ▸; Tickle & Moss, 1999 ▸). Diffuse scattering maps predicted by models of motion can be calculated using either an all-atom covariance matrix or the equation 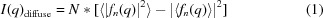
(often called Guinier’s equation, where *q* is the scattering vector, *n* is the complex structure factor of the *n*th protein conformation and *N* is the number of unit cells in the crystal; Phillips *et al.*, 1980 ▸; Micu & Smith, 1994 ▸; Lindner & Smith, 2012 ▸). The covariance matrix describes correlated displacements between every pair of atoms, whereas Guinier’s equation models diffuse scattering from an ensemble of structure factors. Calculation of the covariance matrix has been used to model crystalline normal modes and TLS parameterization (Riccardi *et al.*, 2010 ▸). It is also possible to explicitly estimate each matrix element from molecular-dynamics trajectories (Meinhold & Smith, 2007 ▸). The size of the covariance matrix scales as the square of the number of atoms, making full matrix calculations expensive to compute for large systems. This poses a significant challenge to quantitative diffuse scattering analysis. For these reasons, a straightforward method that calculates diffuse scattering from discrete multi-model PDB files may be preferable.

To meet this need, we developed *phenix.diffuse*, a new tool within the *PHENIX* software suite (Adams *et al.*, 2010 ▸) which uses Guinier’s equation to calculate diffuse scattering from multi-model (ensemble) PDB files. Thus, *phenix.diffuse* can be applied to any motional model represented as an explicit ensemble of related structures. As a first application, we have simulated the diffuse scattering produced by alternative TLS refinements of the glycerophosphodiesterase GpdQ (Jackson *et al.*, 2007 ▸). GpdQ is found in *Enterobacter aerogenes* and contributes to the homeostasis of the cell membrane by hydrolyzing the 3′–5′ phosphodiester bond in glycerophos­phodiesters. Each chain of the dimeric enzyme contains three distinct structural elements: an αβ-sandwich fold containing the active site, a domain-swapped active-site cap and a novel dimerization domain comprised of dual-stranded antiparallel β-sheets connected by a small β-sheet. Although the catalytic mechanism of GpdQ is similar to other metallo-phospho­esterases, some substrates are too large to pass through the active-site entrance as it is modeled in the crystal structure. Protein dynamics must therefore play a role in substrate entry and product release. Normal-mode analysis of the GpdQ hexamer suggested high mobility in the cap domain and a breathing motion centered on the catalytic and dimerization domains (Jackson *et al.*, 2007 ▸). Owing to the high global *B* factors and the presence of diffuse signal in the diffraction images (Fig. 1 ▸ *c*), Jackson and coworkers performed three separate TLS refinements to model the crystalline disorder. All three TLS refinements improved the *R*_free_ values when compared with the standard isotropic *B*-factor refinement; however, there was no significant difference among the final *R*_free_ values from the refinements initiated with distinct TLS groupings. In contrast, our results reveal significant differences between the diffuse intensities predicted by the motion from each TLS refinement, highlighting the possible usefulness of diffuse scattering in optimizing structure refinement.

## 2. Methods   

### 2.1. GpdQ refinement   

Based on the original refinement strategy of Jackson *et al.* (2007 ▸), we performed three different TLS refinements on the zinc-bound structure of GpdQ (PDB entry 2dxn): ‘entire molecule’, with one TLS group for all residues, ‘monomer’, with one TLS group for each of the two individual chains, and ‘sub-domain’, with one TLS group for each of the αβ-sandwich domain (residues 1–196), the ‘dimerization’ domain (residues 197–255) and the ‘cap’ domain (residues 257–271) of each chain. The pre-TLS refinement *R*_work_ and *R*_free_ were 19.1 and 23.1%, respectively. After defining the TLS groups, each structure was re-refined for five macrocycles in *phenix.refine*. The strategy included refinement of the individual coordinates and isotropic *B* factors, water picking and refinement of TLS parameters for defined TLS groups. Both the X-ray/atomic displacement parameters and X-ray/stereochemistry weights were optimized (Afonine *et al.*, 2012 ▸). The final *R*_work_ and *R*_free_ values for each refinement were 14.6 and 18.9% for ‘entire molecule’, 14.9 and 19.0% for ‘monomer’ and 14.9 and 19.3% for ‘sub-domain’, suggesting approximately equal agreement with the Bragg data (Fig. 1 ▸ *d*).

In TLS refinement, the eigenvalues of the T and L matrices describe the variance of the motional displacement along each orthogonal real-space axis. To avoid an unphysical description of TLS motion (Urzhumtsev *et al.*, 2015 ▸), we inspected the eigenvalues of each TLS refinement to ensure non-negative eigenvalues for the T and L matrices (Supplementary Table S1). Although solvent is expected to contribute significantly to experimental diffuse scattering, we removed water molecules after refinement. This step, along with the removal of bulk solvent from the starting structure, ensures that all subsequent diffuse scattering simulations only reflect correlated motions implicit in the TLS refinement.

### 2.2. *phenix.tls_as_xyz* and TLS ensemble generation   

**Figure 2:**
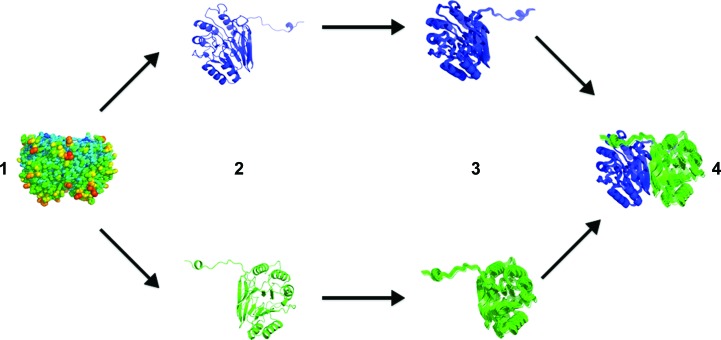
Overview of *phenix.tls_as_xyz*. The input PDB file (1) is broken down into its constituent TLS groups (2) and TLS ensembles are generated for each group independently (3). These groups are then re-assembled into the complete protein structure on a model-by-model basis (4).

We used *phenix.tls_as_xyz* (Urzhumtsev *et al.*, 2015 ▸) to convert the TLS matrices to a structural ensemble. *phenix.tls_as_xyz* receives as input a structure with TLS header information, separates the molecule into individual TLS groups and randomly samples the real-space distribution for each group based on mathematical decomposition of the T, L and S matrices. The trace of the S matrix is set to 0 during these calculations. The sampled PDB files are then either re-assembled into a multi-model PDB ensemble or output with no further changes (Fig. 2 ▸). To ensure adequate sampling of the underlying Gaussian distributions, we generated ensembles of different sizes and monitored the convergence of the global correlation coefficient between diffuse maps in which spherically symmetric sources of diffuse scattering have been removed (‘anisotropic maps’; Supplementary Table S2). These maps offer an improved comparison relative to the raw diffuse signal because they correct for the resolution dependency of diffuse scattering, which would otherwise lead to an overestimation of inter-map correlation. We determined that an ensemble size of 1000 models was sufficient for effective sampling of each TLS refinement. The extent of the motions predicted by the ‘sub-domain’ refinement (Supplementary Fig. S1) is quite surprising and is likely to result from a lack of chemical restraints within the TLS refinement implementation in *PHENIX*. While subdividing the ‘monomer’ TLS refinement into smaller components might intuitively produce similar refinement statistics, the tensors between all three groups are substantially different and thus describe dissimilar motions.

### 2.3. *phenix.diffuse*   

**Figure 3:**
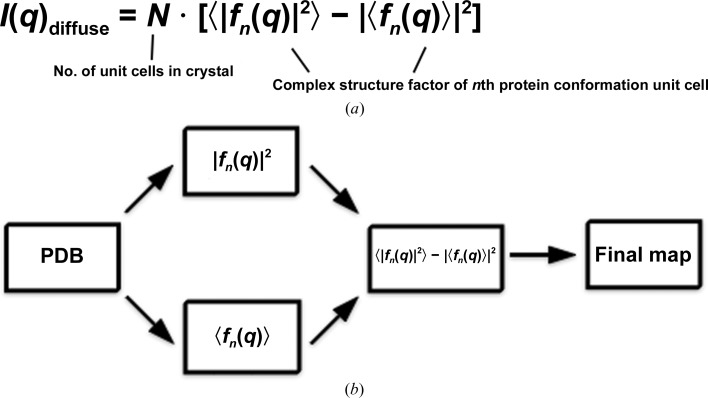
Overview of *phenix.diffuse*. (*a*) The general form of Guinier’s equation. The motion to be analyzed is captured in a series of ‘snapshots’ defined by the the multi-model PDB file. (*b*) The general program flow. Each term in Guinier’s equation is calculated separately from the structural ensembles and then combined to obtain the final map.

*phenix.diffuse* implements Guinier’s description of diffuse scattering (Guinier, 1963 ▸; Fig. 3 ▸ *a*). Diffuse scattering is calculated entirely from a series of unit-cell ‘snapshots’ contained in a multi-model PDB ensemble and assumes no motional correlation between crystal unit cells. This simplification ignores sources of disorder spanning multiple unit cells, which can contribute to experimentally measured diffuse scattering (Doucet & Benoit, 1987 ▸; Clarage *et al.*, 1992 ▸; Wall, Clarage *et al.*, 1997 ▸). *phenix.diffuse* can model these large-scale effects through the analysis of a ‘supercell’ containing multiple unit-cell copies, as implemented in several recent MD simulations of small proteins (Janowski *et al.*, 2013 ▸; Kuzmanic *et al.*, 2014 ▸). Guinier’s equation can be applied to arbitrarily sized crystalline regions; thus, a system of multiple unit cells allows analysis of motions that occur between and across unit cells. In line with previous diffuse scattering simulations (Wall, Van Benschoten *et al.*, 2014 ▸), our program calculates structure factors for each ensemble member at the Bragg lattice positions, from which each term in Guinier’s equation is determined.

### 2.4. GpdQ TLS diffuse scattering simulation   

We simulated the diffuse scattering of each of the GpdQ TLS ensembles to 3.0 Å resolution. Unless otherwise stated, all TLS groups within a given refinement were assumed to move independently of one another. Since the diffraction data for GpdQ in PDB entry 2dxn extend to 2.9 Å resolution, our simulation should be sufficient for future comparisons with experimental maps. As the resulting diffuse scattering data are identical in format to descriptions of Bragg X-ray reflections, *phenix.reflection_statistics* was used to perform all statistical analyses. All reported correlation values are global Pearson correlation coefficients calculated between the described two sets of diffuse intensities. As previously mentioned (and described in Wall, Ealick *et al.*, 1997 ▸), spherically symmetric sources of diffuse scattering contribute significantly to the observed intensity. In order to remove these confounding effects, we used the *LUNUS* software package (Wall, 2009 ▸) to subtract the average radial diffuse intensity from each point (Supplementary Fig. S2).

### 2.5. GpdQ diffraction image processing and radial averaging   

Diffraction images used to determine the GpdQ Bragg structure were collected at the Advanced Photon Source, Lemont, Illinois, USA at cryogenic temperature with 0.25° oscillation wedges (Jackson *et al.*, 2006 ▸). Subsequent processing was performed using *LUNUS* (Wall, 2009 ▸). Pixels correlating to the beamstop shadow and CCD detector panels were removed with the *LUNUS* *punchim* and *thrshim* routines. Solid-angle normalization and beam polarization were corrected using *polarim* and *normim*. Mode filtering was applied as described previously (Wall, Ealick *et al.*, 1997 ▸). The radial intensity profile was calculated from a single image using the *avgrim* function, which calculates radial intensities on a per-pixel scale. The radial profile for the experimental GpdQ data was scaled by a factor of 1000 to better facilitate qualitative comparisons to the simulations.

## 3. Results   

### 3.1. Diffuse scattering is dependent on TLS grouping   

**Figure 4:**
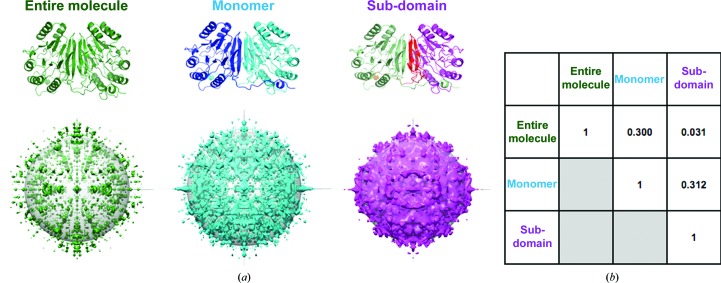
Differing TLS groups produce unique diffuse scattering. (*a*) The GpdQ TLS groups projected onto the structure, along with the calculated diffuse scattering (looking down the *L* axis; the gray sphere denotes 4 Å resolution). The ‘monomer’ and ‘sub-domain’ maps are shown at equivalent density thresholds, while ‘entire molecule’ map is set at 60% of the density threshold. No correlation is assumed between TLS rigid-body groups. (*b*) Pearson correlation coefficients between anisotropic maps.

**Figure 5:**
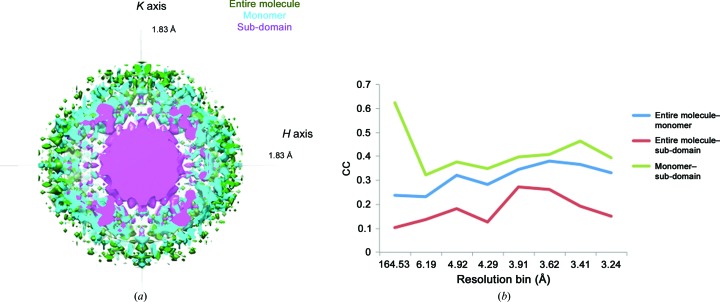
Comparison of simulated GpdQ TLS diffuse scattering maps. (*a*) Cross-section of simulated TLS diffuse scattering maps. Primary and secondary diffuse intensity shells, separated by a gap, can be observed in each model. As the number of TLS groups increase, the intensity shells grow closer, predominantly owing to an expansion in primary intensity shell size. (*b*) Pearson correlation values between each set of maps across resolution bins.

The raw diffuse intensity predicted by the motions described from each TLS refinement strategy rises as a function of the number of TLS groups (Fig. 4 ▸). The ‘entire molecule’ and ‘monomer’ maps show a similar range of intensity values: 0–4.52 × 10^6^ and 0–8.34 × 10^6^, respectively. The ‘subdomain’ map displays a much wider dynamic range (0–4.71 × 10^8^; Supplementary Fig. S1*c*). This trend is likely to result from an increase in the amplitude of TLS motion, particularly within the dimerization region of the ‘subdomain’ model (Supplementary Fig. S1 ▸). However, ‘sub-domain’ map intensities greater than 1 × 10^7^ are limited to a resolution range of 11 Å and lower. The ‘entire molecule’ and ‘monomer’ maps also possess ‘primary diffuse shell’ regions surrounding the origin, although they only extend out to a resolution range of 30 Å. This region will be particularly difficult to measure experimentally given the presence of a beamstop, which blocks access to signal around *F*_000_ (Lang *et al.*, 2014 ▸). Each diffuse map has a dip in radial intensity between the primary diffuse shell before the diffuse intensity increases in a second shell (Fig. 5 ▸ *a*). In contrast to the ‘sub-domain’ map, the strongest diffuse intensities for the ‘entire molecule’ and ‘monomer’ maps occur within this secondary shell. The width between the primary and secondary diffuse shells decreases as the number of TLS groups increases owing to an expansion in the primary diffuse shell radius. As X-ray detectors can easily measure intensities in the regions of reciprocal space occupied by the secondary shell, a significant fraction of the diffuse scattering predicted by TLS refinement can potentially be compared with experimental data.

To determine whether the different TLS groupings yielded distinct diffuse scattering predictions, we calculated the global Pearson correlation coefficient between the anisotropic signal in each refinement . The comparison revealed little similarity between maps (CC in the range from 0.031 to 0.312; Fig. 3 ▸). Comparing the correlation values across resolution bins reveals that the anisotropic diffuse signal correlations remain consistently poor across scattering-vector length (Fig. 5 ▸ *c*). The large discrepancy between the maps calculated with different TLS models contrasts with the high similarity of experimental maps of anisotropic diffuse signal from different crystals of staphylococcal nuclease (CC = 0.93; Wall, Ealick *et al.*, 1997 ▸). This result suggests that the experimentally measured diffuse signal will be sufficiently precise to distinguish between TLS-related diffuse scattering models (Wall, Adams *et al.*, 2014 ▸). However, other sources of disorder will need to be accounted for before models of TLS motion can be effectively compared with experimental data.

### 3.2. Correlations between TLS groups can be detected by diffuse scattering   

**Figure 6:**
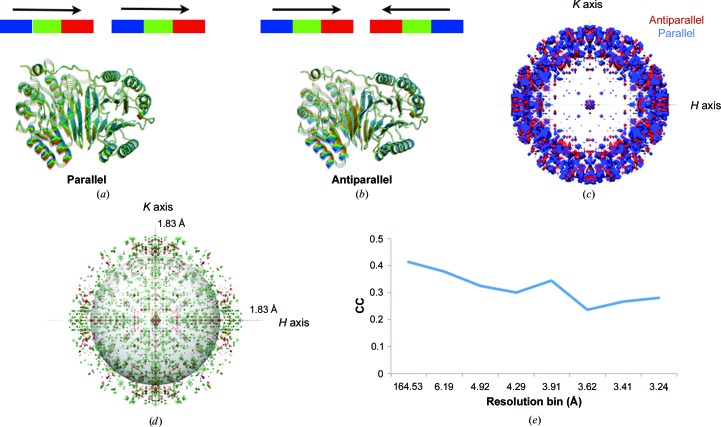
Different correlations between TLS groups produce unique diffuse scattering. Parallel (*a*) and antiparallel (*b*) TLS motions in GpdQ chains result in measurable differences between diffuse scattering patterns (CC = 0.375). Color bars indicate the directionality of the TLS motions; each color represents a unique molecular position. (*c*) A map cutaway reveals strong secondary-shell features with a small primary diffuse shell (looking down the *L* axis; the gray sphere denotes 4 Å resolution). (*d*) Intensity differences between raw ‘antiparallel’ and ‘parallel’ diffuse maps (green, positive; red, negative) highlights the qualitative changes caused by alternative TLS-group correlations. (*e*) Correlation values across anisotropic map resolution bins reveal that the highest correlation occurs between the maps at low resolution and decreases as a function of scattering-vector length.

Although TLS refinement makes no assumptions regarding motion between groups, diffuse scattering can test whether correlated rigid-body fluctuations do, in fact, exist. To illustrate this concept, we simultaneously sampled the motions along the translation and libration eigenvectors to produce ‘parallel’ and ‘antiparallel’ correlated motions for the ‘monomer’ GpdQ TLS refinement (Fig. 6 ▸). For the ‘parallel’ model, the correlated motion consists of sampling along all translation and libration eigenvectors in step sizes of σ/2, where σ is obtained from the underlying Gaussian distribution in each direction, for a total of ten steps (−2.5σ to 2.5σ). Simply reversing the direction of sampling for the chain *B* translation eigenvectors created the ‘antiparallel’ motion. In contrast to the simulation in Fig. 4 ▸(*a*), which assumed no correlation between TLS groups, here we have introduced correlated motion between GpdQ monomers. Next, we simulated the diffuse scattering produced by the ‘parallel’ and ‘antiparallel’ correlated motions. Both raw maps display strong secondary-shell characteristics in combination with a weak primary shell of diffuse scattering (Fig. 6 ▸ *c*). A diffuse intensity difference map (Fig. 6 ▸ *d*) shows that discrepancies between the raw maps occur across the entirety of reciprocal space. Comparing the anisotropic diffuse intensity correlation across resolution bins reveals a general decreasing trend as the scattering-vector length increases (Fig. 6 ▸ *e*). In contrast to the previous TLS simulations, the correlation values are highest at low resolution. The low global Pearson correlation coefficient (0.375) demonstrates that there are quantitative differences between the two maps. However, these intergroup correlation differences will be slightly more difficult to detect than changes between specific TLS models, where the correlation coefficients range from 0.031 to 0.312.

### 3.3. TLS models yield unique radial profiles of diffuse intensity   

**Figure 7:**
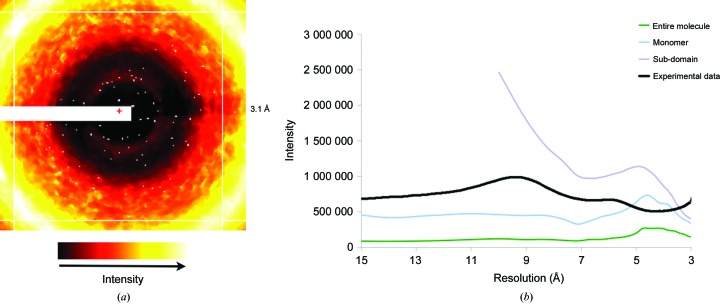
TLS models yield unique radial profiles of diffuse intensity. (*a*) Mode-filtered GpdQ diffraction image used for radial intensity calculation. The white regions correspond to pixels thrown out owing to detector-panel and beamstop artifacts, as well as Bragg scattering contamination. (*b*) Radial diffuse intensity profiles for experimental and simulated GpdQ data. Resolution data below 15 Å (roughly corresponding to the primary diffuse shell) were removed for more accurate visual comparison. The ‘sub-domain’ map exceeds the limits of the *y* axis at lower than 10 Å resolution.

We calculated the radial diffuse intensity profile for a GpdQ diffraction frame and for the three TLS refinements (Fig. 7 ▸). Although radial averaging removes the rich directional information present in diffuse scattering, this simplification has been successfully used to assess agreement between distinct diffuse maps (Meinhold & Smith, 2005 ▸, 2007 ▸). For the experimental GpdQ map, a peak at 8.5 Å and a shoulder at 6 Å are observed. None of these features are observed in the raw TLS radial profiles, except for a local maximum at 4.5 Å and a shoulder at 4 Å for the ‘monomer’ refinement. Rather, the dominating feature for each TLS simulation is the secondary diffuse scattering shell, which varies between maps in both width and maximum radial value. This result is not surprising, as the experimental diffuse scattering from GpdQ reflects a much broader group of correlated motions than simply TLS-related movement within the macromolecule. For example, disordered solvent is expected to significantly contribute to experimental diffuse measurements (Wall, Ealick *et al.*, 1997 ▸). As solvent molecules were not modeled in our TLS ensembles, this is a likely source of the discrepancy between the GpdQ experiment and simulation. The liquid-like motions (LLM) model, in which atoms interact only with nearest neighbors to produce a gelatinous crystalline environment, can also be used to explain the diffuse scattering intensity. Comparing the diffuse maps of staphylococcal nuclease (Wall, Ealick *et al.*, 1997 ▸), pig insulin (Caspar *et al.*, 1988 ▸) and hen egg-white lysozyme (Clarage *et al.*, 1992 ▸) with LLM models maximized correlations across distances of 6–10 Å. Thus, a more thorough analysis involving several models of disorder must be applied to GpdQ to improve the fit to the experimental diffuse data.

### 3.4. Distinct patterns of diffuse signal can be calculated at non-Bragg indices   

**Figure 8:**
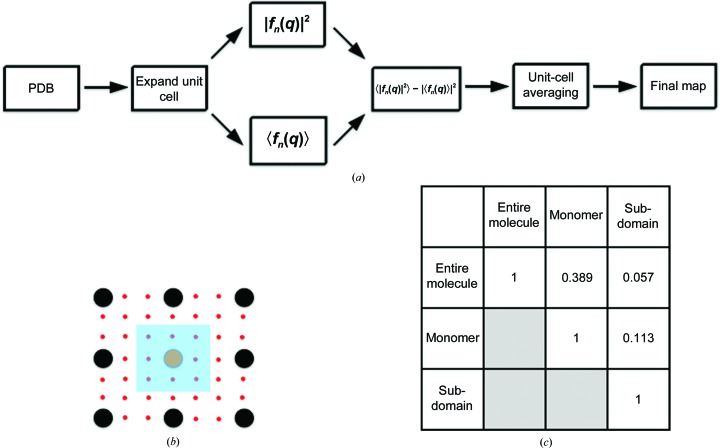
Unit-cell expansion allows reciprocal-space subsampling. (*a*) The unit cell of the input PDB entry is expanded to create the desired unit-cell sampling, each term in Guinier’s equation is calculated separately and then the second term is subtracted from the first to obtain the diffuse intensity. The ‘pseudo-unit cells’ are then averaged across, producing the final diffuse scattering map. (*b*) Unit-cell expansion allowing for 3× subsampling of reciprocal space. True/‘pseudo’ Bragg peaks are shown in black/orange and red, respectively. The intensity values of the eight pseudo peaks and one orange peak in the blue box are averaged and the resulting value is assigned to the Bragg index of the orange peak. (*c*) Pearson correlation coefficients between maps.

While *phenix.diffuse* currently calculates the diffuse signal under Bragg peaks, diffuse scattering occurs throughout the entirety of reciprocal space. To more completely sample reciprocal space between the Bragg spots, we increased the unit-cell boundaries. Expanding the unit cell in real space allows a finer sampling of the underlying Fourier transform (Fig. 8 ▸). The resulting structure factors can be rescaled to the original lattice points, leading to fractional *hkl* sampling. These fractional values are then assigned to the nearest integer *hkl* index and averaged, leading to a single diffuse intensity value associated with each Bragg peak. Although it is clearly possible to output a map consisting of these fractional values and thereby produce a more accurate picture of diffuse scattering, we chose the integer values because diffuse scattering processing techniques commonly calculate the average diffuse intensity across pixels within a 1 × 1 × 1 voxel around each Bragg point (Wall, 1996 ▸). This average value is then assigned to the *hkl* index, leading to the same 1:1 correlation between lattice points and diffuse intensity values. Although it is tempting to use this method in our current analysis, the unit-cell expansion method does not maintain the expected crystallographic symmetry for any crystal system with a screw axis. Introducing vacuum into our structure-factor calculations will satisfy other symmetry operations, but as GpdQ possesses a screw axis we are currently unable to more finely sample its predicted diffuse scattering. Therefore, we can use this method to compare data between simulated models of motion, but not between simulated models and experimental data. More advanced simulation methods will need to incorporate screw axes, either by defining a new supercell for simulation or directly calculating structure factors at fractional *hkl* indices. Cognizant of these limitations, we calculated the diffuse scattering of each of the GpdQ TLS ensembles to 3.0 Å resolution in a *P*1 cell, with a subsampling of 4 × 4 × 4 around each Bragg lattice point (Fig. 8 ▸ *c*). These calculations confirm that each TLS motion produces distinct patterns of diffuse signal throughout reciprocal space.

## 4. Discussion   

Accurate modeling of conformational dynamics is important for understanding macromolecular function. Although many models may fit the existing data equally well, they can often suggest different correlated motions. Our results indicate that comparisons to experimental diffuse scattering can break the degeneracy between different TLS refinements, as different selections of rigid bodies (along with their associated correlations) can produce markedly different diffuse patterns. For example, alternative correlations between TLS groups have equivalent average electron density, but result in unique diffuse scattering predictions. More generally, any model proposed through TLS refinement should agree with the experimental diffuse data, as these data directly reflect the existing protein disorder (Moore, 2009 ▸).

Despite this synergy between TLS refinement and diffuse scattering, there are many potential complications when applying TLS X-ray refinement to model protein dynamics. As the T and L matrices describe independent translations and librations, these motions must be physically sensible. Our review of protein structures deposited in the Protein Data Bank indicates that roughly 85% of refinements employing TLS (about 25% of the total PDB) do not satisfy this physical requirement (Urzhumtsev *et al.*, 2015 ▸). We hypothesize that this discrepancy arises owing to a lack of restraints applied to refined TLS parameters to ensure their physical plausibility. Even if this criterion is met, current TLS refinement methods still do not impose chemical restraints between TLS groups, which can lead to displacements that are chemically unreasonable. Our TLS refinement of the GpdQ subdomain is one such example, as it produces rigid-body displacements that extend across the entirety of the unit cell (Supplementary Fig. S1*c*). Thus, validation checks of TLS refinement (such as those implemented in *phenix.tls_analysis*) are critical, as is employing TLS refinement within a broader framework of restraints. Alternative techniques, such as the phase-integrated method (PIM), which derives anisotropic *B* factors from low-frequency normal modes (Chen *et al.*, 2010 ▸), may significantly improve the biochemical accuracy of modeling efforts. In PIM, the fit between the model and experiment is significantly improved by calculating normal modes in the context of the asymmetric unit rather than individual molecules (Lu & Ma, 2013 ▸).

Numerous sources of crystalline disorder combine to produce observed diffuse intensity patterns. Perhaps the most critical step in diffuse signal analysis is the determination of the relative contribution from each source; *phenix.diffuse* represents an important step towards performing such an investigation. Many causes of disorder can be described in terms of structural ensembles; thus, our tool enables the diffuse scattering produced by each source to be calculated. As experimental diffuse intensity is simply the sum of its independent components, optimizing the relative weights of the hypothesized sources of disorder to best fit the observed diffuse scattering may provide a feasible method of comprehensive diffuse scattering analysis.

With the increasing availability of modeling tools, the lack of high-quality three-dimensional data sets is now a key bottleneck in diffuse scattering analysis. One challenge in data collection is that long X-ray exposures can be required to reveal diffuse features. This can lead to ‘blooming’ around saturated Bragg spots in diffraction images collected using commercially available charge-coupled device (CCD) area detectors (Gruner *et al.*, 2002 ▸). Blooming can artificially increase pixel values between the Bragg spots, where the diffuse intensity is measured (Glover *et al.*, 1991 ▸). Although CCD detectors can be configured to eliminate spot blooming at the cost of decreasing dynamic range (Wall, 1996 ▸; Wall, Ealick *et al.*, 1997 ▸), this configuration is not available in commercial detectors. The development of pixel-array detectors, which possess much higher dynamic ranges as well as very small point-spread functions, has opened the door to more accurate measurement of diffuse signal. Additionally, methods for processing diffuse scattering data from raw image frames to complete reciprocal-space map are under active development (Wall, Adams *et al.*, 2014 ▸). Because acoustic scattering is maximized at Bragg peaks (Glover *et al.*, 1991 ▸), diffuse signal will be most straightforward to measure in intervening regions. These methods will be applied to new data sets of simultaneous Bragg and diffuse scattering data. Instead of being included in the background corrections in estimating Bragg peak intensities, these diffuse intensities will increase the data available for refinement, enable more accurate quantification of interatomic distances (Kuzmanic *et al.*, 2011 ▸) and allow the simultaneous refinement of multiple coupled protein motions (Wilson, 2013 ▸).

## Supplementary Material

Supporting Information.. DOI: 10.1107/S1399004715007415/rr5095sup1.pdf
