# Supplementary material for: Predicting X-ray diffuse scattering from translation–libration–screw structural ensembles
Source: Acta Crystallogr D Biol Crystallogr. 2015 Jul 28;71(Pt 8):1657–67. doi: 10.1107/S1399004715007415 (PMC4528799; doi:10.1107/S1399004715007415)
Supplement: Supplementary file 1 [file d-71-01657-sup1.pdf]

BIOLOGICAL
CRYSTALLOGRAPHY

Volume 71 (2015)

Supporting information for article:

Predicting X-ray diffuse scattering from translation–libration–screw structural ensembles

Andrew H. Van Benschoten, Pavel V. Afonine, Thomas C. Terwilliger, Michael E. Wall, Colin J. Jackson, Nicholas K. Sauter, Paul D. Adams, Alexandre Urzhumtsev and James S. Fraser

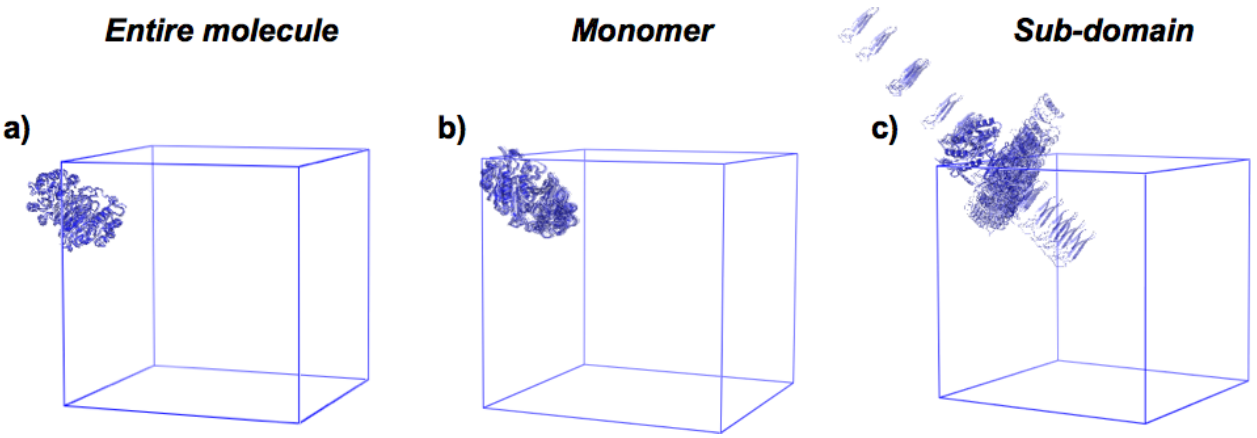

Figure S1 Structural ensembles of GpdQ TLS motions. Each TLS PDB ensemble is shown as a single asymmetric unit outlined by the unit cell. An increase in overall motion is apparent going from left to right. The 20-member ensemble is shown for visual simplicity. It is important to note that the chemically unreasonable motion produced by the sub-domain TLS refinement is not immediately apparent from the T and L eigenvalues presented in Supplemental Table 1, highlighting the need for the more thorough matrix analysis presented in our accompanying paper.

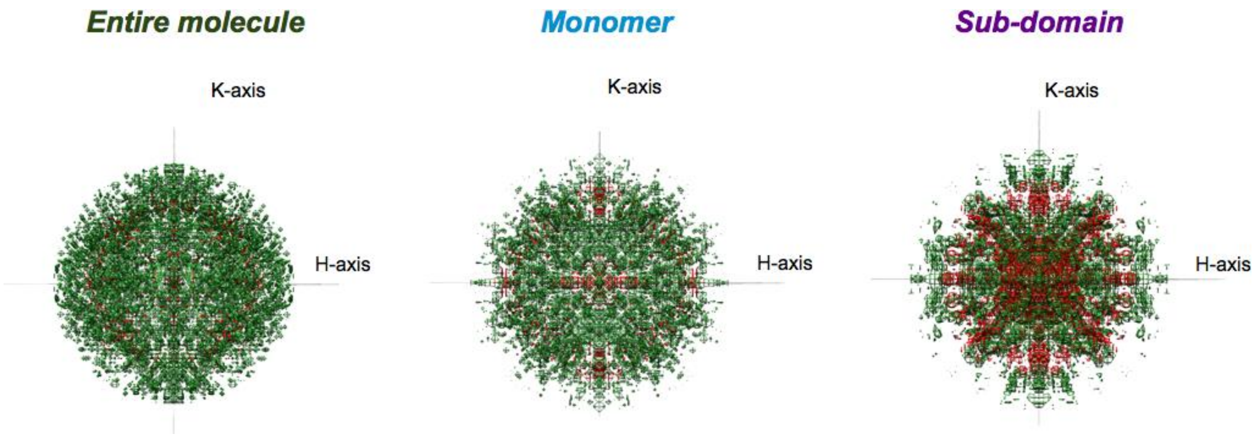

Figure S2 Anisotropic diffuse scattering maps. Positive and negative anisotropic density is shown as green and red mesh, respectively. Absolute threshold levels shown for the positive and negative signals are equivalent. The maps are shown to their full 3Å resolution limit.

Table S1 Eigenvalues of GpdQ TLS refinement matrices.

(a) “Entire molecule”.

T	L
0.854	1.405
0.258	0.717
0.338	0.172

(b) “Monomer”.

T Monomer A	L Monomer A	T Monomer B	L Monomer B
0.873	1.843	0.850	1.896
0.236	0.021	0.192	1.103
0.327	0.822	0.329	0.500

(c) “Sub-domain”. It is important to note that, for the “Sub-domain” refinement, L#5 and L#6 have negative eigenvalues. Due to their low absolute value, however, these eigenvalues were set to zero for subsequent calculations.

T Sandwich (A)	L Sandwich (A)	T Dimerization (A)	L Dimerization (A)	T Cap (A)	L Cap (A)
0.917	0.005	0.942	1.420	0.902	0.154
0.247	1.396	0.313	0.181	0.475	0.062
0.367	0.957	0.375	0.057	0.323	0.005

T Sandwich (B)	L Sandwich (B)	T Dimerization (B)	L Dimerization (B)	T Cap (A)	L Cap (A)
0.940	0.216	0.897	1.07	0.938	0.155
0.170	1.265	0.267	0.059	0.638	**-0.003**
0.399	0.838	0.368	**-0.001**	0.477	0.031

Table S2 Multi-model ensembles are necessary for adequate random sampling of TLS motions

Two ensembles independently sampling the underlying TLS distributions were used to generate anisotropic diffuse scattering maps. Global CC values between the two maps are shown. These simulations were conducted in triplicate, producing the CC standard deviation shown in parentheses. All maps were simulated to 3Å resolution.

	10	***50***	***100***	***500***	***1000***
**Entire Molecule**	0.886 (0.027)	0.956 (0.019)	0.988 (0.005)	0.996 (0.002)	0.999 (0.000)
**Monomer**	0.809 (0.087)	0.924 (0.008)	0.952 (0.005)	0.992 (0.002)	0.997 (0.001)
**Sub-domain**	0.944 (0.012)	0.984 (0.005)	0.992 (0.001)	0.999 (0.000)	0.999 (0.000)
